# Two Cases of Contact Anterior Cruciate Ligament Rupture Combined with a Posterolateral Tibial Plateau Fracture

**DOI:** 10.1155/2015/250487

**Published:** 2015-07-08

**Authors:** Liangjun Jiang, Haobo Wu, Shigui Yan

**Affiliations:** Orthopaedics, The 2nd Affiliated Hospital, Medical College, Zhejiang University, Jiefang Road, No. 88, Hangzhou, Zhejiang, China

## Abstract

*Background.* The combined occurrence of ACL rupture with a posterolateral tibial plateau fracture has not yet been reported. Two cases of such injuries have been treated in our department for the past three years.* Findings. *The two patients both suffered injuries from traffic accidents. The radiological examinations showed a ruptured ACL with fracture of the posterolateral tibial plateau. Reconstruction of the ACL was performed via a standard anatomical single bundle ACL reconstruction technique with autologous tendon by arthroscopy. A posterolateral tibia plateau approach was used to reduce and fix the fractured area with the aid of lag screws. After a one-year follow-up, the two patients recovered well and physical examinations showed full knee range of motion with no evidence of ACL instability.* Conclusions.* The cause of this type injury of ACL rupture with a posterolateral tibial plateau fracture was thought to be by a violent internal tibial rotation/anterior tibial translation without any valgus or varus knee force mechanism during the accident. Satisfactory clinical results were achieved with a standard anatomical single bundle ACL reconstruction by arthroscopy and ORIF for the posterolateral plateau fracture. Both patients reported excellent knee function and fracture healing.

## 1. Introduction

Anterior cruciate ligament (ACL) rupture is frequently associated with other injuries, such as medial collateral ligament (MCL) [1]. However, only a few cases of patients with ACL injury and Segond fracture have been published with an additional osseous injury to the femoral condyle, Gerdy tubercle, or tibial plateau [2]. In particular, the combined occurrence of ACL rupture with a posterolateral tibial plateau fracture, which seems to be caused by some specific mechanisms, has been reported rarely. Two cases of such specific injury (ACL rupture and a posterolateral tibial plateau fracture) from traffic accidents have been treated in our department for the past three years.

## 2. Cases Reports

### 2.1. Patients History

The first case (patient one) was a 29-year-old male and the second case (patient two) was a 38-year-old male patient. Patient one was crushed to a barrier when he was driving a motorbike and patient two was knocked down by a car when he was riding a bicycle. Based on the recollection of the patient history, an axial load on a semiflexed knee with a rotational component was applied to patient's leg. There were no other injuries to these patients.

### 2.2. Physical Examination

Swelling and effusion were observed on the knees. The alignment of the knee was normal. Lachman test was positive (grade II, no end point), the posterior drawer test was negative in both patients, and the valgus and varus instability test at 0° and 30° of the knee flexion was also negative.

### 2.3. Imaging Evaluation

**Figure 1:**
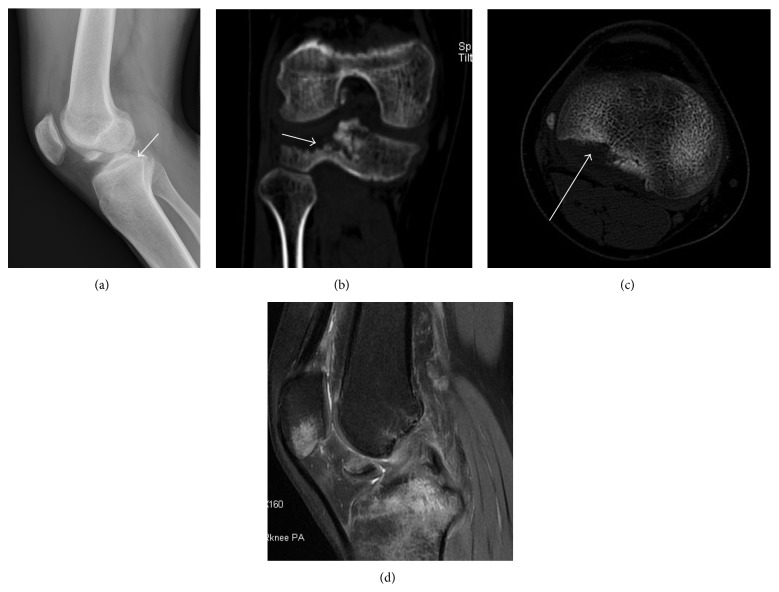
Imaging examinations of the first patient. The X-ray image (a) shows a posterolateral tibial plateau fracture (white arrow). In CT scans (b and c), a posterolateral tibial plateau fracture is found (white arrow). In the MRI image (d), a tortuous ACL was confirmed with the patella and tibia had a bone bruise. No patella fracture was found. There was a fracture fragment in the notch.

**Figure 2:**
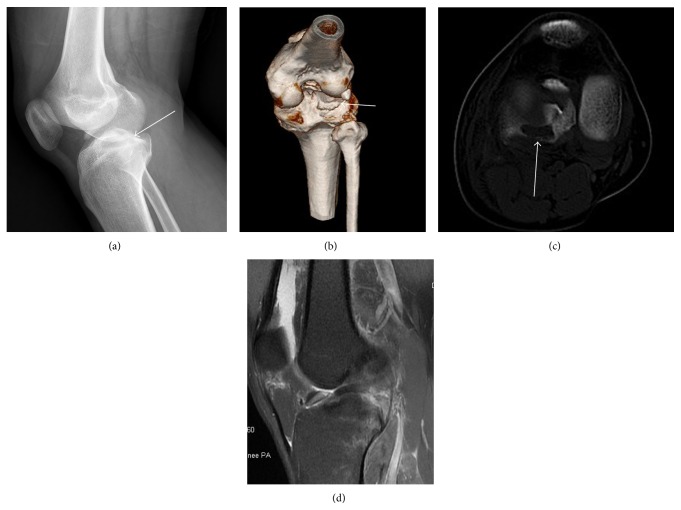
Imaging examinations of the second patient. The X-ray image (a) shows a posterolateral tibial plateau fracture (white arrow). In the CT scans (b and c), a cavity was formed at the lateral location of posterolateral tibial plateau fracture (white arrow). In the MRI image (d), ACL rupture and lateral meniscus tear were confirmed. Other ligaments were not found by the MRI scan.

Plain radiographs and CT revealed a posterolateral tibial plateau fracture. MRI images showed a ruptured ACL, posterolateral tibial plateau fracture (Figures 1 and 2).

### 2.4. Surgical Treatment

**Figure 3:**
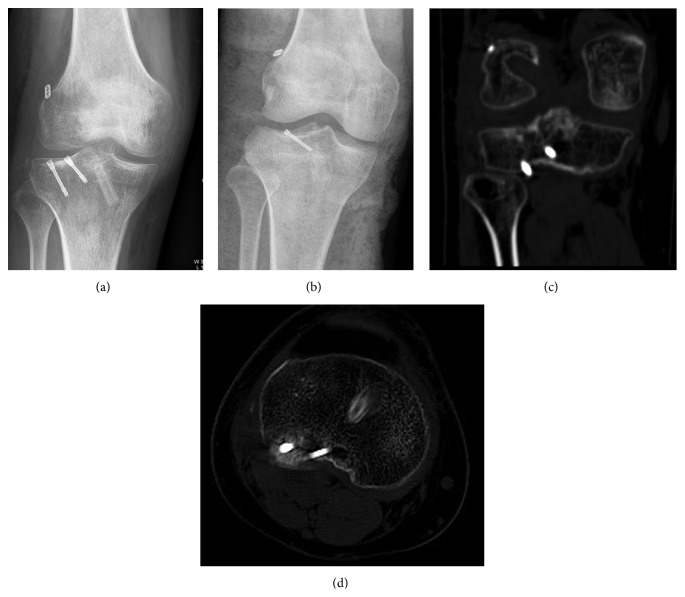
X-ray images of the knees after the operation. In the first patient (a), button steel was used at the femoral site for ACL reconstruction and two lag screws were used for posterolateral tibial fracture fixation. In the second patient (b), similar procedure to that for the first patient, but only one screw, was used. (c and d) The CT scans in patient one showed the tibial plateau fracture was well reduced and fixed.

In these patients, ACL reconstructions were performed via a standard anatomical single bundle ACL reconstruction technique with autologous hamstring tendon by arthroscopy. A posterolateral tibia plateau approach was used for the fixation of lag screws for the posterolateral plateau fracture (Figure 3). The fibular nerve and popliteus tendon should be carefully protected during the approach. The fractures were easily reduced and the screws were inserted into the cartilage.

### 2.5. Follow-Up

The follow-up was 15 months in patient one and 12 months in patient two. The two patients experienced no symptoms of pain and no joint effusion on their knee joints and were all capable of performing daily activities. There were no reports of instability and both patients could do moderate sports. Physical examinations showed no evidence of ACL laxity (both grade 0 in Lachman classification), full knee range motion (0° to 130°), and negative anterior drawer, pivot shift, and posterior drawer tests. X-ray images showed bone healing at fracture site. The IKDC 2000 score was 80.5 in patient 1 and 83.9 in patient 2 [3].

## 3. Discussion

This study reports the occurrence of a rare knee injury by car accident, ACL contact rupture with a posterolateral tibial fracture. Several combined injuries with ACL rupture have been reported. The most common injury published is the MCL/ACL injury. Another common injury is the complex knee ligament injuries, which are characterized by simultaneous rupture of the ACL and/or the posterior cruciate ligament and at least one collateral ligament [4]. Segond fracture is associated with ACL injury for 75% to 100% of the time, and it occurs in approximately 9% to 12% of all ACL tears [5]. Since its original description in 1878, Segond fracture has been defined as a small elliptic avulsion fracture of the proximal lateral tibial. It represents a rupture of the lateral capsular fibers of the iliotibial tract or anterior oblique band of the lateral collateral ligament [6]. This type of injury has its specific mechanism by tibial internal rotation with varus force placed on the middle portion of the lateral capsule and the associated meniscotibial ligament (by internal rotation of the tibial with flexed knee) [7]. The impact between the posterior aspect of the lateral tibial plateau and the lateral femoral condyle causes these small avulsion fractures during the knee-joint injury [8]. Tei et al. [9] reported a combined osteochondral fracture of the posterolateral tibial plateau and Segond fracture with ACL injury in a skeletally immature patient. In their opinion, the possible injury mechanism was collision of the posterior border of the lateral tibial plateau and the lateral femoral condyle as a result of abnormal internal rotation (subluxation) of the knee joint at the time of injury. According to Speer et al. [10], in most of the ACL injuries, the knees experience a subluxation with an osseous contusion between femur condyle and tibial plateau. If the knee subluxation is slight, then only some bone bruises occur. By contrast, if the knee subluxation caused by accident is violent enough, then damage similar to that in Tei et al. and in our patients may occur. In our patients, the knees went through subluxation and internally rotated during the accident. MRI images showed grade I medial compartment bone bruise which supported the combined internal tibial rotation/anterior tibial translation mechanism. The possible injury mechanism in our patients was similar to the combined internal tibial rotation/anterior tibial translation mechanism in noncontact ACL injury. However, our patients were specific. Only a posterolateral tibial plateau fracture combined with ACL rupture was found and with some bone bruises, no other injuries were observed. Thus, the impact between the lateral femoral condyle and posterolateral tibial plateau was violent but very centralized that only one area was fractured. We hypothesized that the posterolateral tibial fracture was stroked by the femoral condyle during the injury when the knee subluxation and internal tibial rotation occurred. Such injury or fracture only occurs in an accident-induced ACL injury for a noncontact ACL injury could not offer so strong but centralized force. Because the MRI images showed no collateral ligaments damage, the knee valgus or varus may not have occurred when injury happened. Therefore, this type of ACL injury could be due to a violent internal tibial rotation or anterior tibial translation without any knee valgus or varus force mechanism during the accident. In patient one, the patella had a big bone bruise in the MRI without any fracture in the CT. We supposed this injury was due to the impact between the patella and barrier because the bone bruise was on the superficial surface of patella not the articular surface.

About the treatment: this type of fracture mainly has two injuries: the ACL rupture and a posterolateral tibial plateau fracture. So, we reconstructed these two structures. The ACL reconstruction was done before the open reduction of the plateau fracture and internal fixation because arthroscopy needed an enclosed articular space. The posterolateral tibial plateau fracture did not make any interference with the ACL reconstruction. The fractures could be seen by arthroscopy and no posterior cruciate ligament damage was found. Since the plateau fractures were too large, it was hard to do the reduction and fixation by arthroscopy. We used the posterior lateral approach to reveal the fracture area. The fibular nerve and popliteus tendon were carefully protected. After the capsule was opened, the fracture could be easily seen and then reduced. Both fractures showed simple fracture, so we used lag screws for absolute stability. At last, we made the anterior drawer shift test and the valgus and varus instability test again to confirm the results for the surgery. According to the examinations before surgery, there were no other ligament injuries and with no other repair being done.

## 4. Conclusion

Two cases of contact rupture of ACL combined with posterolateral tibial plateau fracture after knee accident were reported. The cause of the injury was thought to be by a violent internal tibial rotation/anterior tibial translation without any valgus or varus knee force mechanism during the accident. In the treatment, a standard anatomical single bundle ACL reconstruction by arthroscopy and ORIF for the posterolateral plateau fracture was performed with satisfactory clinical results. Both patients reported excellent knee function and fracture healing.
